# Clinical predictors of all‐cause mortality in patients presenting to specialist heart failure clinic with raised NT‐proBNP and no heart failure

**DOI:** 10.1002/ehf2.12742

**Published:** 2020-06-04

**Authors:** Pankaj Garg, Steven Wood, Andrew J. Swift, Graham Fent, Nigel Lewis, Dominic Rogers, Alexander Rothman, Athanasios Charalampopoulos, Abdallah Al‐Mohammad

**Affiliations:** ^1^ Department of Infection, Immunity & Cardiovascular Disease University of Sheffield Sheffield UK; ^2^ Sheffield Teaching Hospitals NHS Foundation Trust Sheffield UK

**Keywords:** Natriuretic peptides, Heart failure, Echocardiography, Left ventricular function, Observational study, Electrocardiography

## Abstract

**Aims:**

Clinical outcomes for patients suspected of having heart failure (HF) who do not meet the diagnostic criteria of any type of HF by echocardiography remain unknown. The aim of this study was to investigate the clinical predictors of all‐cause mortality in patients with suspected HF, a raised N‐terminal pro‐b‐type natriuretic peptide (NTproBNP) and who do not meet the diagnostic criteria of any type of HF by echocardiography.

**Methods and results:**

Relevant data were taken from the *S*heffield *HEA*rt *F*ailure (SHEAF) registry (222349P4). The inclusion criteria were presence of symptoms raising suspicion of HF, NTproBNP > 400 pg/mL, and preserved left ventricular function. Exclusion criteria were any type of HF by echocardiography. The outcome was defined as all‐cause mortality. Cox proportional‐hazards regression model was used to investigate the association between the survival time of patients and clinical variables; 1031 patients were identified with NTproBNP > 400 pg/mL but who did not have echocardiographic evidence of HF. All‐cause mortality was 21.5% (222 deaths) over the mean follow‐up (FU) period of 6 ± 2 years. NTproBNP was similar in patients who were alive or dead (*P* = 0.96). However, age (HR 1, *P* < 0.01), chronic kidney disease (CKD, HR 1.2, *P* < 0.01), chronic pulmonary obstructive disease (COPD, HR 1.6, *P* < 0.01), dementia (HR 5.9, *P* < 0.01), male gender (HR 1.4, *P* < 0.01), first‐degree atrioventricular block (HR 2.1, *P* < 0.01), left axis deviation (HR 1.6, *P* = 0.04), and diabetes (HR 1.4, *P* = 0.03) were associated with all‐cause mortality. In multivariate regression, age, gender, CKD stage, COPD, and dementia were independently associated with mortality. In patients with NTproBNP > 627 pg/mL, NYHA class predicted death (II, 19.6%; III, 27.4%; IV, 66.7%; *P* < 0.01).

**Conclusions:**

Patients with no HF on echocardiography but raised NTproBNP suffer excess mortality particularly in the presence of certain clinical variables. Age, male gender, worsening CKD stage, presence of COPD, and dementia are independently associated with all‐cause mortality in these patients. An NTproBNP > 627 pg/mL coupled with NYHA class could identify patients at greatest risk of death.

## Introduction

The prevalence of heart failure (HF) is rising,^1^ mainly because of a significant increase in HF with preserved ejection fraction (HFpEF)^2^ and the ageing population. In addition, survival after a diagnosis of HF has shown only modest improvement in the last decade.^3^ Many of the symptoms and clinical signs of HF are non‐specific, hence, the need for additional tests to confirm that symptoms are caused by structural and/or functional abnormalities of the heart. Routinely, N‐terminal pro‐b‐type natriuretic peptide (NTproBNP) and transthoracic echocardiography (TTE) are used to confirm the diagnosis of HF. While NTproBNP is mainly used as a screening test for HF and a prognostic tool in HF, TTE is used to detect structural and functional indicators suggestive of HF and its underlying causes.^4^, ^5^

Those patients presenting to the HF diagnostic clinic with suspicion of HF and rise of the NTproBNP >400 pg/mL, whose echocardiograms do not meet the diagnostic criteria of any of the types of HF are thought not to have HF. We know that the rise of NTproBNP is associated with worsening prognosis even if the patient does not have HF.^6^ It would be of clinical importance to characterise the group of patients with raised NTproBNP who are deemed not to have HF. Moreover, there is particular anxiety related to the rise in the incidence and prevalence of HFpEF, especially given the fact their diagnosis is dependent on indirect markers of the rise of the left ventricular end‐diastolic pressure. In other words, one needs to ensure that no one with HFpEF is misdiagnosed as not having HF. The clinical profile of patients with HFpEF remains varied and is evolving over time. Moreover, it remains unclear if patients who do not seem to meet the criteria of HFpEF by current methods have any significant increase in long‐term mortality.

This study sought to investigate the clinical profile and outcomes of patients presenting with symptoms or signs that may suggest HF, a raised NTproBNP and no echocardiographic evidence of HF.

## Methods

### Data and patients

Patients in Sheffield with a suspected diagnosis of HF from a population of 550 000 are screened by primary care physicians using a single measurement of NTproBNP. In accordance with the National Institute of Health and Care Excellence (NICE) guidelines those with a positive NT‐proBNP and symptoms or signs that may suggest HF are referred to the diagnostic HF clinic in Sheffield, UK.^7^, ^8^ Data are collected prospectively and electronically encrypted with an annual data validation check. The analytical cohort was derived from all patients presenting to the regional HF unit between 13 April 2012 and 28 November 2018. Inclusion criteria for this study were the following: age 18 years or over, raised NTproBNP (>400 pg/mL) and preserved LV function. Exclusion criteria included any type of HF including HFpEF, HF secondary to a mid range reduction in EF (HFmEF) or HF with reduced EF (HFrEF), significant valvular heart disease or pulmonary hypertension. Patients who did not have full echocardiographic study were excluded from this study.

### Heart failure assessment

All patients referred to the HF clinic from primary care had NTproBNP equal to or higher than 400 pg/mL. All patients underwent a resting 12‐lead electrocardiogram and a TTE examination. After these tests, each patient was clinically assessed by a HF specialist. The final diagnosis was determined by the responsible HF specialist according to the presenting history, clinical examination, and the results of investigations in keeping with NICE chronic HF guidelines.^7^, ^9^

### Echocardiography

Echocardiography was performed as per the consensus statement. Both the clinical physiologist performing the echocardiogram and the treating cardiologist were involved to make the final integrated diagnosis of a form of HF or no evidence of HF if the left ventricle was preserved, and there was no evidence of raised LV filling pressure.

### Research ethics

The *S*heffield *HEA*rt *F*ailure registry (SHEAF registry) has been sanctioned by the local 3D lab committee under the registration number—222349P4. This has the appropriate research ethics committee approval (17/YH/0142). This study complies with the Declaration of Helsinki.

### Study variables

Clinical variables were extracted from the regional electronic HF database: patient demographics (age, sex, and date of first contact with HF services), past medical history [systemic hypertension, diabetes, ischaemic heart disease, hyperlipidaemia, smoking status, valve disease, chronic obstructive pulmonary disease (COPD), peripheral vascular disease, pulmonary embolus, prior coronary or valvular intervention, dementia, and stroke], and clinical symptom burden defined by New York Heart Association (NYHA) functional status and presence of any angina. Renal functional stratification was made using the chronic kidney disease (CKD) stages on renal biochemistry tests and recorded in the HF database^10^. NTproBNP was recorded in all patients. The NTproBNP assay used was the Roche NT‐Pro BNP assay. CV is 5% at concentrations of 130 and 4000.

The 12‐lead electrocardiogram documented rhythm (sinus, atrial fibrillation, or paced), QRS duration, left axis deviation, ST/T‐wave changes, presence of ectopic beats, any conduction delays or atrioventricular blocks (AVB), and presence of LV hypertrophy using the standard voltage criteria^11^. Similarly, details of the echocardiographic findings were registered in the database.

### Statistical analysis

Statistical analyses were performed in IBM SPSS version 25.0, 64 bits and MedCalc version 19.0.5. Categorical baseline characteristics are described with numbers and percentages. Continuous variables are described using means and standard deviation (SD). The outcome was defined as all‐cause mortality. All continuous variables were compared to death using independent sample *T*‐test. Categorical variables were compared using *χ* ^2^ *T*‐test. Initially, the unadjusted association of mortality was calculated in a univariate logistic regression model, and results presented as hazard ratios [HRs, 95% CI (confidence interval)]. Forest plot was used to visualise the significance of individual clinical variables. A subgroup total was calculated as the average of relative risks and CIs to check for overall significance. The univariable *χ* ^2^ values tested the strength of these associations and permitted benchmark comparisons between clinical measures in models where higher values equate stronger associations and lower *P* values. Subsequently, the Cox proportional‐hazards regression model was used to investigate the association between the survival time of patients and clinical variables significance in univariate analysis (*P* < 0.05). Kaplan–Meier curves are used to visualise and interpret the data of variables associated with mortality. Kaplan–Meier curves used the log‐rank test to investigate the differences in curves between alive and dead patients at FU. Finally, c‐statistics determined the threshold of NTproBNP for its association to mortality in patients using the Youden index criterion value from the receiver operating curve (ROC) analysis investigating its association to all‐cause mortality. The NTproBNP threshold was then used to investigate the association of HF symptom burden defined by NYHA functional class and all‐cause mortality. The Kruskal–Wallis one‐way ANOVA test was used to investigate the overall differences in mortality rates between different NYHA functional classes. All tests were two‐sided, and statistical significance was considered *P* < 0.05.

## Results

### Patient characteristics

Baseline characteristics of the cohort are summarised in *Table* *1*. We identified 1031 patients who presented to the regional HF services with raised NTproBNP with normal LV filling pressure on TTE, normal contraction, and no valvular abnormalities with no sign of pulmonary hypertension or right ventricular impairment. The mean age of the study population was 76.5 ± 9 years, and 55.5% were women. The average NTproBNP was 939.5 ± 865 pg/mL. From the total cohort, 34% (*n* = 352) patients were in AF. The AF patient population had significantly higher NTproBNP levels when compared to non‐AF patients (1192 ± 827 pg/mL vs. 797 ± 852 pg/mL, *P* < 0.05). The average follow‐up time was 6 ± 2 years for all patients. The all‐cause mortality in our study was 21.5% (222 deaths). From the whole cohort, 47% were on β‐blockers, 38% on angiotensin converting enzyme inhibitors, 4% on mineralocorticoid antagonists, and 3% on diuretics.

**Table 1:** Demographics of the study population sub‐categorised depending on mortality at 6‐year follow‐up

	Alive	Dead	*P*‐value
% (N)	78.5 (809)	21.5 (222)	
Age (years)	76 ± 9	79 ± 8	<0.01
NT‐pro BNP (pg/mL)	939 ± 866	942 ± 863	0.96
Male % (*N*)	42 (343)	52 (116)	<0.01
12 lead electrocardiographic findings			
Sinus rhythm % (*N*)	61 (493)	76 (169)	<0.01
Atrial fibrillation % (*N*)	37 (303)	22 (49)	
Paced % (*N*)	2 (13)	2 (4)	
QRS < 120 msec % (*N*)	93 (749)	90 (199)	0.23
QRS = 120–150 msec % (*N*)	5 (44)	9 (19)	
QRS > 150 msec % (*N*)	2 (16)	2 (4)	
Left axis deviation % (*N*)	5 (41)	9 (20)	0.02
Ectopic beats % (*N*)	2 (19)	3 (7)	0.49
1st‐degree AVB % (*N*)	4 (31)	9 (21)	<0.01
Higher degree AVB % (*N*)	1 (6)	1 (2)	0.81
T‐wave inversion % (*N*)	5 (41)	6 (13)	0.64
ST‐segment changes % (*N*)	4 (29)	6 (13)	0.13
Voltage criteria of LV hypertrophy % (*N*)	3 (23)	4 (9)	0.35
Symptoms burden			
NYHA I % (*N*)	30 (246)	34 (75)	0.05
NYHA II % (*N*)	59 (478)	51 (114)	
NYHA III % (*N*)	10 (81)	13 (29)	
NYHA IV % (*N*)	1 (4)	2 (4)	
Angina % (*N*)	14 (113)	15 (34)	0.26
Risk factors for heart disease			
CKD 0% (*N*)	41 (331)	29 (64)	0.01
CKD 1% (*N*)	9 (69)	11 (24)	
CKD 2% (*N*)	15 (122)	16 (35)	
CKD 3% (*N*)	33 (264)	39 (87)	
CKD 4% (*N*)	2 (20)	4 (9)	
CKD 5% (*N*)	0 (3)	1 (3)	
Ischaemic heart disease % (*N*)	25 (200)	25 (56)	0.87
Valvular heart disease % (*N*)	7 (55)	10 (23)	0.07
Systemic Hypertension % (*N*)	62 (498)	63 (140)	0.68
Diabetes % (*N*)	14 (115)	20 (44)	0.04
Hypercholesterolaemia % (*N*)	17 (136)	14 (30)	0.24
Smoker % (*N*)	28 (223)	24 (54)	0.33
COPD % (*N*)	18 (147)	28 (63)	<0.01
Dementia % (*N*)	0 (2)	4 (8)	<0.01
Stroke % (*N*)	10 (82)	14 (31)	0.11

### Association of clinical parameters to all‐cause mortality

As expected, during follow‐up, mortality was higher in more elderly patients (*P* < 0.01) (*Table* *1*). NTproBNP at presentation was similar in the no‐morality and mortality group (*P* = 0.96). A greater number of female patients presented to HF clinic; however, male patients had greater all‐cause mortality (25.3% vs. 18.5%, *P* < 0.01).

Of the 13 recorded ECG parameters, three parameters were significantly different between those with all cause mortality and those who survived. First‐degree atrioventricular block (AVB) and left axis deviation were more common in patients with all cause mortality (9% vs. 4% [*P*<0.01] and 9% vs. 5% [*P*=0.02], respectively). Whereas AF was less common in patients with all cause mortality (14% vs. 26%, *P*<0.05).The latter remained the case even when we looked at patients with AF and higher NTproBNP levels (>797pg/mL) than non‐AF patients (14.1% mortality vs. 25.5% mortality, *P* <0.001).

Advancing CKD stage was also associated with a rise in all‐cause mortality. Among the other co‐morbidities diabetes mellitus type 2, COPD and dementia were more prevalent in patients with all‐cause mortality.

### Regression

**Figure 1:**
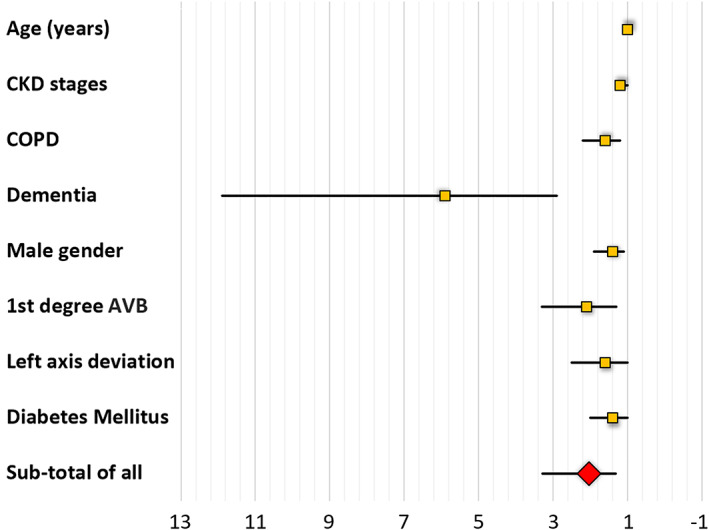
Forest plot demonstrating relative risk (orange box) and its 95% CI (black line) of clinical variables that demonstrated association with all‐cause mortality (*P* < 0.05). The subtotal relative risk was 2 (95% CI: 1.3–3.3).

Univariate regression identified eight variables associated with all‐cause mortality—age, advancing CKD stages, history of COPD, history of dementia, male gender, first‐degree AVB, left axis deviation, and history of diabetes mellitus (*Table* *2*, *Figure* *1*). Multivariate cox proportional‐hazards regression identified five variables with independent association to all‐cause mortality—age, advancing CKD stages, history of COPD, history of dementia, and male gender, of which dementia was the most closely related to mortality (24.8, HR 6.1; 95% CI: 3–12.6).

**Table 2:** Cox proportional‐hazards regression of all the clinical variables which had significant association to all‐cause mortality

	Univariate model			Multivariate model		
	*χ* ^2^ value	HR (95% CI)	*P*‐value	*χ* ^2^ value	HR (95% CI)	*P*‐value
Age (years)	29.3	1 (1–1.1)	<0.001	23	1 (1–1.1)	<0.001
CKD (Stages 0–5)	11.1	1.2 (1–1.3)	<0.001	5.3	1.12 (1–1.2)	0.02
COPD*	10.7	1.6 (1.2–2.2)	0.001	13	1.7 (1.3–2.3)	<0.001
Dementia*	24.1	5.9 (2.9–11.9)	<0.001	24.8	6.1(3–12.6)	<0.001
Gender	6.9	1.4 (1.1–1.9)	0.009	8.7	1.5 (1.1–1.9)	0.003
1st‐degree AVB*	10.1	2.1 (1.3–3.3)	0.001	‐	‐	‐
Left axis deviation*	4.1	1.6 (1–2.5)	0.04	‐	‐	‐
Diabetes mellitus*	4.7	1.4 (1–2)	0.03	‐	‐	‐

The single asterisks denote the clinical variables that showed significant association with all cause mortality as shown on the Forest plot in Figure 1.

**Figure 2:**
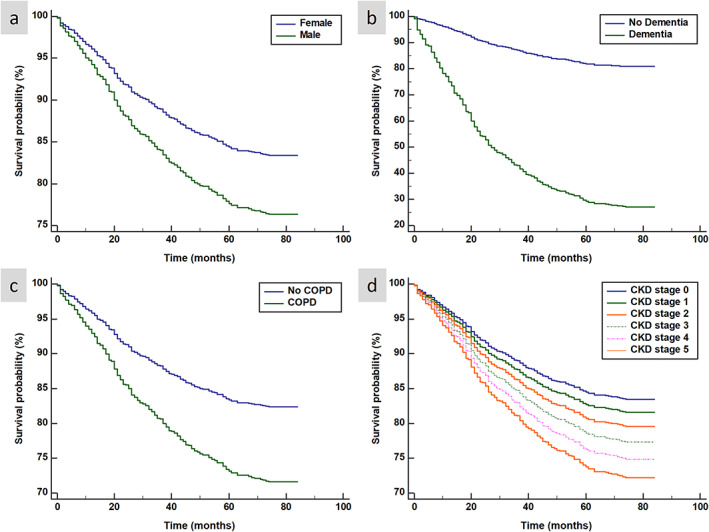
Kaplan–Meier (KM) plots for the risk of all‐cause mortality (*n* = 222) in the SHEAF registry of 1031 patients with raised NTproBNP and no evidence of HFpEF on TTE. Panel (a) demonstrates the KM plots for gender and the significantly increased incidence of mortality in men. Panel (b) demonstrates increased mortality with worsening CKD stages. Panel (c) demonstrates COPD is associated with worsening mortality. Panel (d) demonstrates that a diagnosis of dementia in this cohort is associated with poorer outcomes.

Kaplan–Meier and log‐rank analysis demonstrated significantly worse survival curves for male patients as well as patients with dementia, COPD, and advancing stages of CKD (*Figure* *2*).

### NYHA burden and mortality

**Figure 3:**
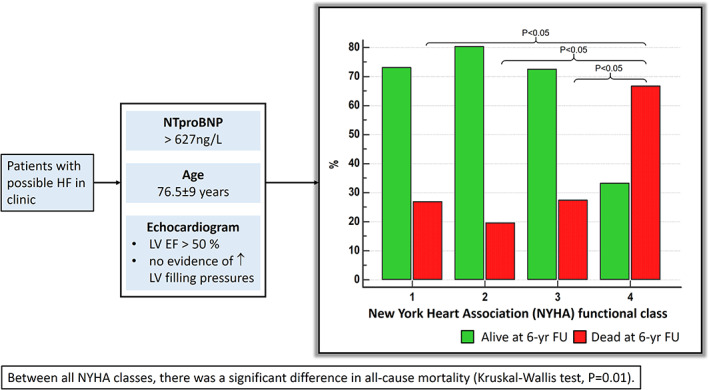
A significant rise in all‐cause mortality with higher symptom burden (NYHA class II‐IV) in patients with no evidence of HF on echocardiography.

The optimum threshold for NTproBNP on c‐statistics was 627 pg/mL for association to mortality in our cohort (AUC:0.51, 95% CI: 0.48 to 0.54; *P* = 0.64). In patients with NTproBNP higher than 627 pg/mL, we noted that worsening NYHA functional class was associated with all‐cause mortality (NYHA II, 19.6% mortality; NYHA III, 27.4% mortality; and NYHA IV, 66.7% mortality; *P*‐value = 0.01) (*Table* *3*). Conover post hoc analysis revealed significant differences within each NYHA symptomatic group (*Figure* *3*).

**Table 3:** In patients with raised NTproBNP over the threshold, increasing HF symptom burden demonstrated association with increasing all‐cause mortality

NYHA class	Alive at FU	Dead at FU	*χ* ^2^ (*P*‐value)	*χ* ^2^ for trend (*P*‐Value)
II	266 (80.4%)	65 (19.6%)	9.2 (*P* = 0.01)	6.7 (*P* < 0.01)
III	45 (72.6%)	17 (27.4%)		
IV	2 (33.3%)	4 (66.7%)		

### NTproBNP and clinical variables associated with all‐cause mortality

As expected from the literature, NTproBNP was significantly higher in males versus females [734 pg/mL, interquartile range (IQR): 1180–513 vs. 639 pg/mL, IQR: 998–481; *P* < 0.001] (see Supporting Information, *Figure* *S1*). Similarly, with advancing CKD stages, there was a significant rise in NTproBNP (*P* < 0.001). In post hoc analysis, NTproBNP levels for CKD Stages 0 to 2 were significantly lower than for CKD Stages 3 to 5 (*P* < 0.05). NTproBNP levels for patients in CKD Stage 1 were significantly lower than the levels for patients in CKD Stages 4 and 5 (*P* < 0.05). NTproBNP levels in patients with CKD Stage 4 were significantly lower than the levels in patients with CKD Stage 5 (833, IQR: 1255–695 vs. 3808, IQR: 10 415–1356, *P* < 0.05).

However, NTproBNP levels was comparable in patients with and without COPD (683 pg/mL, IQR: 965–502 vs. 663 pg/mL, IQR: 1122–492; *P* = 0.64) and those with or without dementia (514 pg/mL, IQR: 703–432 vs. 668 pg/mL, IQR: 1102–494; *P* = 0.14).

### Discussion

In this study, we demonstrate that patients with suspected HF who present with raised NTproBNP, but whose echocardiogram does not suggest the presence of any form of HF including HFpEF, have high all‐cause mortality over a mean FU period of 6 years. Clinical factors which were associated with all‐cause mortality included age, male gender, left‐axis deviation, first‐degree heart block, advancing CKD stages, history of diabetes mellitus, COPD, and dementia. However, clinical factors which were independently associated with all‐cause mortality were age, male gender, CKD stage, COPD, and dementia. It was noteworthy that using a higher threshold of NTproBNP of 627 pg/mL, worsening NYHA function class was associated with poorer outcomes. This study highlights that these patients warrant further clinical attention and calls for potential future studies to investigate if they truly do not have raised LV filling pressures that may define HFpEF.

The prognostic role of NT‐proBNP in the general population remains controversial. However, a recent meta‐analysis of eight studies investigating the role of NT‐proBNP for prognostication in the general population, concluded that NT‐proBNP levels are associated with all‐cause mortality^12^. In the eight studies which reported all‐cause mortality in the general population, over an average follow‐up period of 7 years, the mortality was 16%. This is less than the mortality we report at 21.5%. Importantly, the authors of the meta‐analysis also noted that the risk of all‐cause mortality was higher in studies with a follow‐up duration ≤5 years than in those with >5 years of follow‐up, suggesting death events mainly occurs in the early follow‐up duration. Our study has many differences to general population‐based studies. Firstly, our patient population only includes patients who have suspected HF because of symptoms or signs and raised NTproBNP who have been referred by primary care physicians for specialist advice. Secondly, our follow‐up period was longer than 5‐years, and this may explain why we did not demonstrate overall significant association between NTproBNP levels and mortality. The question remains as to whether some of these patients may still develop HF at a later stage and that the initial assessment despite their symptoms or signs could not detect any echocardiographic evidence of HF of any type.

The mortality rate for HFpEF from previously published studies remains unacceptably high—32% at 3–4 years follow‐up. In our study, we demonstrate that the all‐cause mortality rate for the people who were not found to have HF was lower at 21.5% for a mean FU period of 6 years. When we looked at patients with the higher threshold of NTpro‐BNP (627 pg/mL), and investigated their all‐cause mortality rates, patients with NYHA III demonstrated a mortality rate of 27.5%, which is not too far from the mortality rate of patients with HFpEF, described in the literature. Importantly, patients with NYHA IV symptoms had more than double the all‐cause mortality rates at 66.7%. This suggests that in patients who do not have evidence of any other type of HF, but have a high burden of symptoms with significantly raised NTproBNP, further tests may be needed, and perhaps careful follow‐up of these patients should be considered given their high risk of events.

Previous studies have shown that cardiovascular magnetic resonance (CMR) can provide tissue characterisation in HFpEF to further sub‐phenotype HFpEF^13^. Kanagala *et al*. made the point that CMR can detect previously undiagnosed pathology by echocardiography in 27% patients. Most importantly, a new CMR diagnosis was the strongest independent predictor of adverse outcome (HR: 1.92; 95% CI: 1.07 to 3.45; *P* = 0.03) in their study.

The mean age of patients in our study is greater than 76 years. Not unexpectedly advancing age independently predicted all‐cause mortality, although the patients who died during up to 6‐year follow up were only 3 years older than patients who were alive (76 vs. 79 years old). Gender strongly influences the outcomes in various cardiovascular diseases^14^. In comparison to our study, general population based studies have reported varying death rate in the elderly population above the age of 70 (20% in early 70s to 35% in late 70s)^15^. Moreover, in the elderly, symptoms of reduced effort tolerance or shortness of breath are often poorly reported,^16^, ^17^ and hence, it remains unclear how many patients from the general population are in the high risk. What is obvious is that the elderly with increased symptom burden, especially shortness of breath, have significantly higher mortality^18^, ^19^.

Recent evidence from epidemiological studies suggests that male patients with HFpEF tend to have a worse clinical course^20^. Similarly, this study demonstrates that male patients who have raised NTproBNP and no evidence of HF on echocardiography, have 8% increase in all‐cause mortality when compared to their female counterparts. Compared with Lam *et al*.'s sub‐study of ‘The Irbesartan in Heart Failure With Preserved Ejection Fraction (I‐PRESERVE)’ Trial which evaluated gender difference in HFpEF, our HR for male gender was slightly higher for all‐cause mortality when compared to their study (1.4 vs. 1.025). Another study by Duca *et al*., demonstrated increased cardiac death but not all‐cause mortality in male patients with HFpEF when compared with their female counterparts. Our study also demonstrates that the overall NTproBNP levels were higher in men at presentation than in women (see Supporting Information, *Figure* *S1*). However, this is not surprising as female gender is known to be associated with lower levels of NTproBNP, when compared with men in the general population.

One of the key negative findings of this study is that NTproBNP alone does not predict mortality in patients who have no evidence of HF by echocardiography. However, when it is taken into account with the symptom burden, assessed as NYHA functional class, it does appear to have a complimentary role. In our study, higher threshold for NTproBNP (>627 pg/mL) and increasing NYHA functional class, were associated with increasing mortality. Hence, patients with higher degree of symptoms should not be ignored and further tests to confirm no HF may be necessary in this patient population.

There is limited literature which has investigated the clinical relevance of ECG changes in HFpEF^21^, ^22^. The study carried out by Cenkerova *et al*. demonstrated that HFpEF patients who died during the course of FU, had a tendency to have a prolonged QTc interval (427 ± 42 ms vs. 454 ± 42 ms, *P* = 0.058)^21^. Another study comparing HFpEF with HF because of reduced ejection fraction (HFrEF) for ECG changes, demonstrated that higher resting heart rate, abnormal P‐wave axis, and abnormal QRS‐T axis were associated with HFpEF^22^. However, this study did not look at mortality as primary end‐point and only compared ECG in the two HF states. Contrary to both these studies, our study investigated the most commonly reported ECG abnormalities. We noted that left axis deviation and first‐degree AVB were the only two ECG markers associated with all‐cause mortality. Left axis deviation has been shown in both population‐based studies^23^ and in acute HF^24^ to be associated with all‐cause mortality. Certainly, its role in cohorts of patients who do not have echocardiographic evidence of any type of HF though they have symptoms suspicious of HF and raised NTproBNP, like our cohort, had not been previously studied.

Our study did not demonstrate an increase in mortality in patients with AF. We suspect this is probably because these patients are reviewed and managed by specialists who further optimised their therapy as required.

Worsening CKD stage is a well‐established marker of poorer outcomes and all‐cause mortality^25^, ^26^. It is also known that the higher the CKD stage, the higher the baseline of NTproBNP would be^27^. NTproBNP is excreted from the kidney in the active form or as metabolites. In case of worsening renal function, NTproBNP levels are likely to go up. This will be even more pronounced in patients who have raised intra‐cardiac pressures. Results from this study demonstrate a similar association of CKD stage and all‐cause mortality in patients with no evidence of HF or raised LV filling pressure. Hence, the rise in all‐cause mortality may be in part related to CKD plus the functional decline identified by NYHA functional class in our study. It is worth noting that at advanced stages of renal impairment (CKD Stages 4–5), NTproBNP rises steeply highlighting the importance of looking at altering the NTproBNP thresholds based on co‐morbidities such as CKD.

### Limitations

First, this observational study includes data from a single centre. However, the SHEAF registry includes diverse data of HF patients from Sheffield in England. Even though the ethnic background of patients was not recorded, this region of England is ethnically very diverse (Asian: 7.3%, black: 1.5%, mixed: 1.6% white: 85.8% and other: 3%)^28^. Second, as this is an observational study, the results of this study are mainly hypothesis‐generating. Further prospective studies are warranted to investigate and ascertain the phenotype of the patients with breathlessness, raised NTproBNP, and normal TTE. Cardiovascular magnetic resonance imaging has demonstrated some promise to better categorise diastolic function^29^, ^30^, ^31^, ^32^, ^33^, ^34^; however, this needs further invasive catheter‐based validation studies. Third, this study did not record the cause of death.

## Conclusion

Symptomatic patients with no HF but raised NTproBNP and normal LV filling pressure on echocardiography suffer excess mortality, particularly in the presence of certain clinical variables. Age, male gender, worsening CKD stage, presence of COPD, and dementia are independently associated with all‐cause mortality in these patients. An NTproBNP>627 pg/mL coupled with NYHA class could identify patients at greatest risk of death. Further studies are needed to better sub‐phenotype this group of patients and determine any underlying aetiology that may warrant further intervention.

## Conflict of interest

None declared.

## Funding

AR is supported by Clinical Research Career Development Fellowships from the Wellcome Trust (206632/Z/17/Z). AS is supported by Wellcome Trust (205188/Z/16/Z). PG is supported by the Academy of Sciences Starter Grant (SGL018\1100).

## Supporting information


**Data S1.** Supporting Information
