# Supplementary figures and images for: Clinical predictors of all‐cause mortality in patients presenting to specialist heart failure clinic with raised NT‐proBNP and no heart failure
Source: ESC Heart Fail. 2020 Jun 4;7(4):1791–800. doi: 10.1002/ehf2.12742 (PMC7373941; doi:10.1002/ehf2.12742)

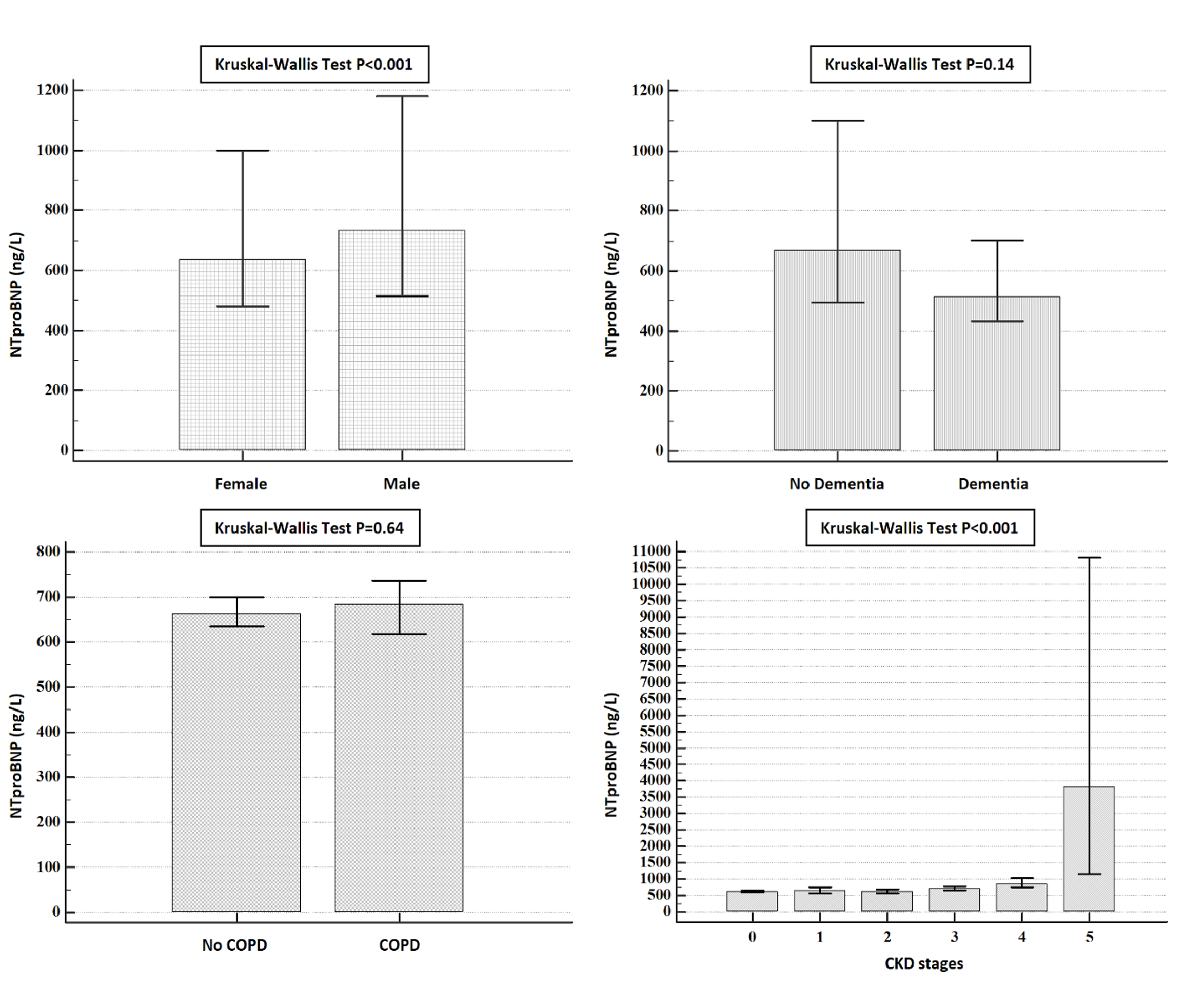

Supplement: Supplementary file 1 — Data S1. Supporting Information [file EHF2-7-1791-s001.tiff]
